# RAD6 inhibition enhances paclitaxel sensitivity of triple negative breast cancer cells by aggravating mitotic spindle damage

**DOI:** 10.1186/s12885-022-10119-z

**Published:** 2022-10-18

**Authors:** Brittany M. Haynes, Kristen Cunningham, Malathy P. V. Shekhar

**Affiliations:** 1 Karmanos Cancer Institute, 4100 John R Street, Detroit, MI 48201 USA; 2 Department of Oncology, Wayne State University School of Medicine, 421 E. Canfield Avenue, Detroit, MI 48201 USA; 3 Present address: Office of Policy Communications, and Education, National Center for Advancing Translational Sciences, Besthesda, USA; 4 Department of Pathology, Wayne State University School of Medicine, 421 E. Canfield Avenue, Detroit, MI 48201 USA

**Keywords:** Paclitaxel, Acquired resistance, BRCA1, RAD6, TAU, Cyclin B1

## Abstract

**Background:**

Paclitaxel (PTX), a first-line therapy for triple negative breast cancers (TNBC) induces anti-tumor activity by microtubule stabilization and inhibition of cell division. Its dose-limiting toxicity and short half-life, however, pose clinical challenges underscoring the need for strategies that increase its efficiency. RAD6, a E2 ubiquitin conjugating enzyme, is associated with centrosomes at all phases of cell cycle. Constitutive overexpression of the *RAD6B* homolog in normal breast cells induces centrosome amplification and multipolar spindle formation, indicating its importance in centrosome regulation.

**Methods:**

TNBC centrosome numbers were scored by pericentrin immunostaining. PTX sensitivities and interactions with SMI#9, a RAD6-selective small molecule inhibitor, on TNBC cell survival were analyzed by MTT and colony forming assays and an isogenic MDA-MB-468 TNBC model of PTX resistance. The molecular mechanisms underlying PTX and SMI#9 induced cytotoxicity were determined by flow cytometry, immunoblot analysis of cyclin B1 and microtubule associated protein TAU, and dual immunofluorescence staining of TAU and α-tubulin.

**Results:**

Our data show aberrant centrosome numbers and that PTX sensitivities are not correlated with TNBC BRCA1 status. Combining PTX with SMI#9 synergistically enhances PTX sensitivities of BRCA1 wild-type and mutant TNBC cells. Whereas SMI#9/PTX combination treatment increased cyclin B1 levels in MDA-MB-468 cells, it induced cyclin B1 loss in HCC1937 cells with accumulation of reproductively dead giant cells, a characteristic of mitotic catastrophe. Cell cycle analysis revealed drug-induced accumulation of tetraploid cells in S and G2/M phases, and robust increases in cells with 4 N DNA content in HCC1937 cells. TAU overexpression is associated with reduced PTX efficacy. Among the six TAU isoforms, both SMI#9 and PTX downregulated 1N3R TAU in MDA-MB-468 and HCC1937 cells, suggesting a common mechanism of 1N3R regulation. Dual TAU and α-tubulin immunostaining showed that SMI#9 induces monopolar mitotic spindles. Using the isogenic model of PTX resistance, we show that SMI#9 treatment restores PTX sensitivity.

**Conclusions:**

These data support a common mechanism of microtubule regulation by SMI#9 and PTX and suggest that combining PTX with RAD6 inhibitor may be beneficial for increasing TNBC sensitivities to PTX and alleviating toxicity. This study demonstrates a new role for RAD6 in regulating microtubule dynamics.

**Supplementary Information:**

The online version contains supplementary material available at 10.1186/s12885-022-10119-z.

## Introduction

The taxane-based drugs paclitaxel (Taxol) and docetaxel (Taxotere) interfere with microtubule (MT) dynamics by stabilizing MTs and are commonly used as first-line chemotherapies for various solid tumors including triple negative breast cancer (TNBC). MTs are composed of α- and β-tubulin subunits that dimerize to promote MT nucleation during mitosis, and this nucleation is facilitated and stabilized by γ -tubulin ring complexes (γ-TuRCs) and interaction with α-tubulin at the centrosome [1, 2]. Taxane-based drugs bind to the β-tubulin subunit of assembled MTs and prevent MT depolymerization which is necessary for completion of mitosis causing mitotic arrest and subsequent mitotic catastrophe [3–6]. While both paclitaxel (hereafter referred as PTX) and docetaxel exert their toxic effects in a similar manner, docetaxel binds more efficiently to β-tubulin than PTX resulting in increased potency [1, 7]. However, this increased potency is associated with increased toxicity when compared to PTX [8]. While taxanes have been shown to improve disease-free survival in combination therapy settings, cytotoxicity and acquisition of resistance are major clinical challenges [1, 9–12].

The microtubule associated protein TAU stabilizes and regulates MT dynamics and its overexpression is associated with PTX resistance as TAU binds to β-tubulin in the same region as PTX and consequently competes with PTX [13, 14]. The Human TAU protein is encoded by the *MAPT* gene on chromosome 17. Expression of *MAPT* results in six major TAU isoforms that vary in the number of N-terminal inserts (0 N, 1 N, or 2 N) and C-terminal repeat domains (3R or 4R) due to alternative splicing of exons 2, 3, and 10, resulting in molecular sizes between 48 kDa (0N3R) and 67 kDa (2N4R) of the corresponding proteins [15]. Both the 3R and 4R isoforms bind to microtubules [16] with the 3R isoforms binding with lower avidity than the 4R isoforms [17].

Besides the role of BRCA1 in DNA damage response and repair, BRCA1 also plays an important role in centrosome function via monoubiquitinations of γ-tubulin, a major component of the ɣ-TuRCs [18–20]. Loss of BRCA1 by mutation or methylation has been linked with taxane resistance [11, 18–22]. Monoubiquitination of ɣ-tubulin at K48 and K344 prevents its recruitment to the centrosome with resultant inhibition of centrosome reduplication and reduction in MT nucleation [23–27]. Deubiquitination of ɣ-tubulin by BAP1 reverses these effects allowing for regulated MT polymerization, depolymerization and centrosome replication. This delicate balance between ɣ-tubulin ubiquitination and deubiquitination is critical for regulation of centrosome number and rates of MT nucleation that is essential for proper chromosome segregation. Therapeutically, regulation of taxane binding and efficiency are influenced by MT polymerization/depolymerization rates and centrosome replication. Thus, BRCA1 wild type cancers are more sensitive to taxanes due to BRCA1 mediated inhibition of MT nucleation and centrosome regulation as compared to BRCA1 mutant cells that have reduced γ-tubulin monoubiquitination, elevated rates of MT polymerization/depolymerization, and centrosome amplification that potentially hinder taxanes from binding to MTs [19, 24, 25, 28–30].

RAD6 is a major component of the translesion synthesis (TLS) or DNA damage tolerance pathway and its E2 ubiquitin conjugating activity is essential for this function [31, 32]. The two closely related human homologs of yeast *RAD6*, *UBE2A* (or *RAD6A*) and *UBE2B* (or *RAD6B*) are localized on chromosomes Xq24-25 and 5q23-31, respectively, and share 95% amino acid identity [33]. We have shown previously that *RAD6B* rather than *RAD6A* is overexpressed in breast cancers and that constitutive overexpression of *RAD6B* in normal breast cells induces transformation and chemoresistance whereas silencing of RAD6B in breast cancer cells compromises TLS activity and renders them chemosensitive [34, 35]. We have also shown that RAD6B is associated with centrosomes at all phases of cell cycle, and in contrast to wild type BRCA1, constitutive overexpression of *RAD6B* in normal human breast cells induces centrosome amplification, multipolar mitotic spindles and chromosomal aneuploidy [34]. RAD6B is overexpressed in TNBC cell lines and clinical tumors independent of their BRCA1 status, and its overexpression correlates with breast cancer progression and chemoresistance [34–37]. TCGA analysis showed TNBC patients with high *RAD6B* expression have 2.34 times shorter overall survival compared to those with low *RAD6B* expression [38]. We have developed a RAD6-selective small molecule inhibitor, SMI#9, and the details of its synthesis, characterization, and in vivo pharmacokinetics and therapeutic activity on TNBCs are described [39, 40]. SMI#9 treatment inhibits MT depolymerization and induces G2/M arrest [39]. Using an isogenic TNBC model of acquired PTX resistance, Kenicer et al. [41], reported gains in chromosome 5q31, a locus harboring *RAD6B*, suggesting the potential involvement of *RAD6B* in taxane resistance. Several mechanisms of PTX resistance have been described including overexpressions of multidrug resistance-1 (MDR-1)/P-glycoprotein (PgP), drug efflux transporter (ABCB1), and CYP-mediated PTX detoxification [42], all of which increase the required dose of PTX with time and in turn produce greater toxicity.

Here we present data that demonstrate an important role for RAD6B in mitotic spindle dynamics and PTX response. Using BRCA1 wild type and mutant TNBC cells, we show that their PTX responses do not correspond with BRCA1 status in the TNBC cell models tested, and that inhibition of RAD6 with SMI#9 enhances PTX sensitivities of TNBC cells. The SMI#9 induced PTX sensitization is associated with reduced expression of TAU isoforms and increases in the proportion of mitotic cells with monopolar mitotic spindles. Cell cycle analysis showed PTX and SMI#9 treatments induced accumulation of tetraploid cells in S-phase and G2/M arrested cells compared to controls in both MDA-MB-468 and HCC1937 cells. Cells with 4 N DNA content (tetraploid G1 state, a feature of mitotic slippage) were also robustly increased by drug treatments in HCC1937 cells compared to controls. Addition of SMI#9 to PTX treatment enhanced cyclin B1 levels in PTX-sensitive MDA-MB-468 TNBC cells whereas PTX and SMI#9 treatments of PTX-resistant HCC1937 TNBC cells induced cyclin B1 loss and accumulation of cells with enlarged or multiple nuclei, a characteristic of mitotic slippage and catastrophe. Using an isogenic MDA-MB-468 model of acquired PTX resistance, we show that SMI#9 treatment restores PTX sensitivity, providing further support for RAD6 role in PTX response.

## Material and methods

### Cell lines and culture

*BRCA1* wild type MDA-MB-468 and MDA-MB-231, and *BRCA1* mutant HCC1937 TNBC cells were purchased from ATCC (Manassas, VA). *BRCA1* mutant SUM1315 TNBC cells were purchased from Asterand (Detroit, MI). All cell lines were maintained in Dulbecco’s Minimal Essential Medium/F12 (DMEM/F12; Invitrogen, Carlsbad, CA) supplemented with 5% fetal bovine serum. After authentication by cell bank (short tandem repeat PCR) and mycoplasma screening (MycoAlert, Lonza), to minimize cell drifting several aliquots of cells were frozen and used within 10–15 passages of obtaining them from the sources described. Isogenic cells resistant to PTX were generated by exposing MDA-MB-468 cells to gradually increasing doses of clinical grade PTX (Hospira, Inc., Lake Forest, IL) starting from 1 nM over four to six months to select MDA-MB-468 cells capable of tolerating at least threefold higher levels of PTX as their parental counterpart. To ensure sustained drug resistance, the generated PTX-resistant (PTX^R^) MDA-MB-468 cells were maintained in media supplemented with 20 nM PTX and are referred as MDA-MB-468-PTX^R^ cells.

### Cell viability assay

MDA-MB-231, MDA-MB-468, SUM1315, HCC1937 or MDA-MB-468-PTX^R^ cells were seeded at a density of 5–7 × 10^3^ cells per well in 96-well plates and treated with vehicle or 0.1–100 nM PTX. To assess the effect of RAD6 inhibition on PTX sensitivity, cells were treated with 0.75 µM SMI#9 alone or together with PTX. Cell viability was assessed at 72 h posttreatment by MTT assays. Experiments were performed in triplicates and results presented are representative of three independent experiments. Phase contrast images were taken at 24–48 h posttreatment with the Olympus IX71 microscope equipped with a Hamamatsu digital camera. PTX and SMI#9 interaction was determined using CompuSyn software (Combosyn Inc., Paramus, NJ, USA) and combination index (CI) values calculated: CI < 1, CI = 1, and CI > 1 indicate synergistic, additive, and antagonistic effects, respectively [43, 44].

### Clonogenic cell survival analysis

For evaluation of colony forming potentials, MDA-MB-468 or HCC1937 cells were treated overnight with PTX (0.75, 1.5 or 4 nM, MDA-MB-468; 5, 10, 25, 50 or 100 nM, HCC1937), and reseeded in drug-free media at 100 cells per well in 24-well plates in triplicates. PTX treated cells were allowed to form colonies comprising ~ 50 cells when they were treated for 72 h with either 0.75 µM SMI#9 or vehicle. Colonies were detected by crystal violet staining and assessed with GelCount™ Oxford Optronix and CHARM algorithm with a minimum diameter of 100 µm set as the threshold for colony classification. Colony composition was determined by the presence of giant or multinucleated cells. Colony forming efficiency was expressed relative to control cells and results were expressed as mean ± S.E.M from at least two independent experiments.

### Immunoblot analysis

MDA-MB-468 or HCC1937 cells were treated with PTX at their respective IC25 and IC50 doses, 0.75 µM SMI#9, or a combination of PTX and 0.75 µM SMI#9, and whole cell lysates were prepared at 48 h as previously described [36]. To analyze the effects of proteasome inhibitor on cyclin B1 regulation, PTX and SMI#9 treated HCC1937 cells were also treated with the 26S proteasome inhibitor MG132 (700 nM). 50–100 µg of protein were subjected to 4–20% gradient SDS-PAGE and western blot analysis of RAD6 [34], TAU (antibody recognizing nonphosphorylated and phosphorylated TAU isoforms, Abcam, Waltham, MA), cyclin B1 (Sigma-Aldrich Chemicals, St. Louis, MO), and β-actin (Sigma-Aldrich Chemicals). Although molecular and gene silencing analysis have established RAD6B as the predominant RAD6 gene overexpressed in breast cancer cell lines and tissues, the RAD6 protein detected by our antibody is referred as RAD6 rather than RAD6B as the peptide we used for RAD6B antibody generation is 91% conserved in human RAD6A and thus fails to distinguish RAD6A and RAD6B proteins [34]. Protein levels relative to the loading control β-actin were quantified by Image J.

### Cell cycle analysis

Cells were synchronized by serum starvation for 72 h prior to treatment with 5 or 10 nM paclitaxel, 0.75 µM SMI#9 or both for 24 or 48 h. Cells were fixed with 70% ethanol, incubated with DNase free RNase A, stained with propidium iodide (PI) and measured using a Northern Lights full spectrum flow cytometer (3-laser configuration; Cytek Biosciences, Fremont, CA) at the Microscopy, Imaging, and Cytometry Resources Core of Karmanos Cancer Institute. An unstained control was used to unmix cellular autofluorescence from PI fluorescence during acquisition using SpectroFlo software (Cytek). Flow Cytometry Standard (FCS) files were exported, then imported into FlowJo (FlowJo LLC, Ashland, OR) where they were gated to remove cell aggregates (based on PI-Height vs PI-Area) and the 2 N DNA peak position was normalized. Gated, normalized FCS files were exported, then imported into ModFit LT v.5.0 (Verity Software House, Topsham, ME) which deconvoluted the DNA histograms using the tetraploid model with debris and apoptosis modeling.

### Immunofluorescence staining

To assess centrosome numbers, MDA-MB-231, MDA-MB-468, SUM1315 or HCC1937 cells were fixed in 10% phosphate buffered formalin, permeabilized with methanol/acetone (1:1, v/v), and immunostained with anti-pericentrin (Abcam, MA) antibody and the corresponding FITC-conjugated secondary antibody (Molecular Probes, Eugene, OR). Nuclei were counterstained with 4’,6-diamidino-2-phenylindole (DAPI). To evaluate the effects of SMI#9 and PTX treatments on TAU localization and MT spindles, MDA-MB-468 or HCC1937 cells were treated overnight with 0.75 µM SMI#9, PTX (2.5 nM, MDA-MB-468 cells; 10 nM, HCC1937 cells) or a combination of 0.75 µM SMI#9 and PTX, and subjected to immunostaining with anti-α-tubulin (Sigma-Aldrich Chemicals, MO) and anti-TAU (Abcam, MA) antibodies and the corresponding Texas Red or FITC-conjugated secondary antibodies, respectively. Nuclei were counterstained with DAPI and scored for TAU-positive cells and polarity of MT spindles. To assess nonspecific reactions, slides were stained in the absence of primary antibody or with isotype matched nonimmune IgG. Images were collected on an Olympus BX40 microscope equipped with a Sony high resolution/sensitivity CCD video camera and processed using CellSens software. The results shown are representative of data collected from at least 40 cells in five-seven fields and from two independent experiments.

### Statistical analysis

Comparisons between two individual groups or across three or more groups were done using two-tailed independent t-test or one-way ANOVA. Experimental results are presented as the mean ± standard error of mean (S.E.M). Statistical significances were considered if *P* < 0.05. All statistical analyses were performed with GraphPad Prism.

## Results

### BRCA1 status does not correlate with centrosome number or PTX response

**Fig. 1:**
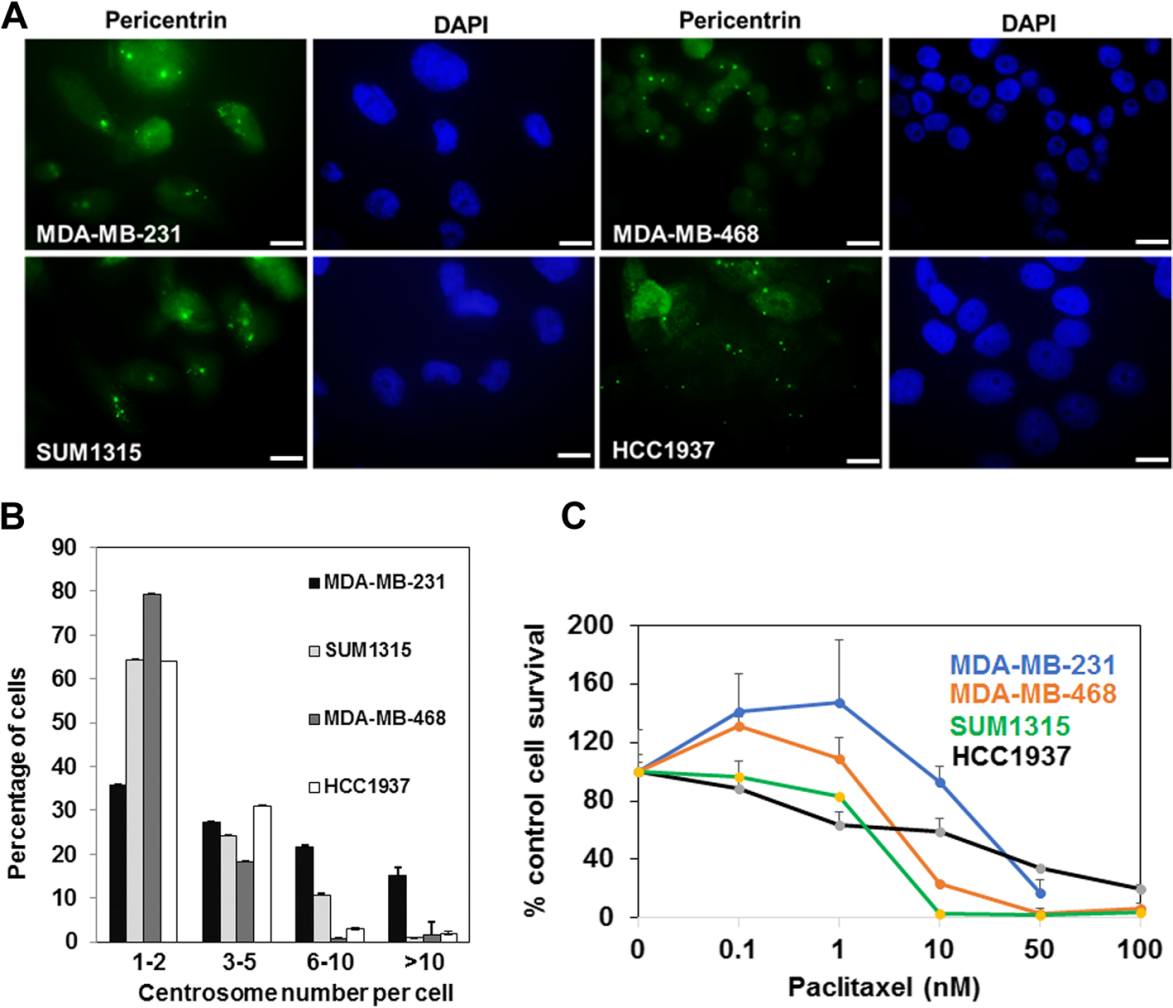
Lack of association between BRCA1 status and centrosome number and paclitaxel (PTX) response.** A** TNBC cell lines were fixed and immunostained with anti-pericentrin antibody. Nuclei were counterstained with DAPI. Bar, 10 µm. **B** Centrosome number quantification. Approximately 75 cells in four to six fields were scored for centrosome number. Data are shown as mean ± S.E.M from at least 2 independent experiments. Bars, 10 µm. **C** PTX sensitivities of BRCA1 wild type MDA-MB-231, MDA-MB-468, and BRCA1 mutant SUM1315 and HCC1937 TNBC cell lines measured by MTT assays. Results are expressed as mean ± S.D. of three independent experiments

Loss of BRCA1 function has been implicated in centrosome amplification and PTX resistance [24, 25, 29]. To determine whether TNBC cells with wild type or mutant BRCA1 display variation in centrosome numbers, TNBC cells were immunostained for the centrosomal protein pericentrin (Fig. 1A and B). All TNBC cell lines showed centrosome amplification with ~ 64% of MDA-MB-231 and ~ 20% of MDA-MB-468 BRCA1 wild type cells, and ~ 35% of SUM1315 and ~ 36% of HCC1937 BRCA1 mutant cells containing ≥ 3 centrosomes (Fig. 1B).

To assess PTX sensitivity of BRCA1 wild type and mutant TNBC cells, MTT assays were performed with MDA-MB-231, MDA-MB-468, SUM1315 and HCC1937 cells exposed to increasing concentrations of PTX (Fig. 1C). The results showed no correlation between the BRCA1 status and PTX sensitivity as the BRCA1 wild type MDA-MB-231 cells (IC50 35 nM) were most resistant followed by BRCA1 mutant HCC1937 cells (IC50 25 nM), BRCA1 wild type MDA-MB-468 (IC50 5 nM) and BRCA1 mutant SUM1315 (IC50 4 nM) cells. However, when separated into TNBC subtypes, PTX responses of the basal subtype (MDA-MB-468 and HCC1937) correlated with their BRCA1 status whereas this association was not observed with mesenchymal subtype (MDA-MB-231 and SUM1315) TNBC cells.

### RAD6 inhibition sensitizes both BRCA1 wild type and mutant TNBC cells to PTX

**Fig. 2:**
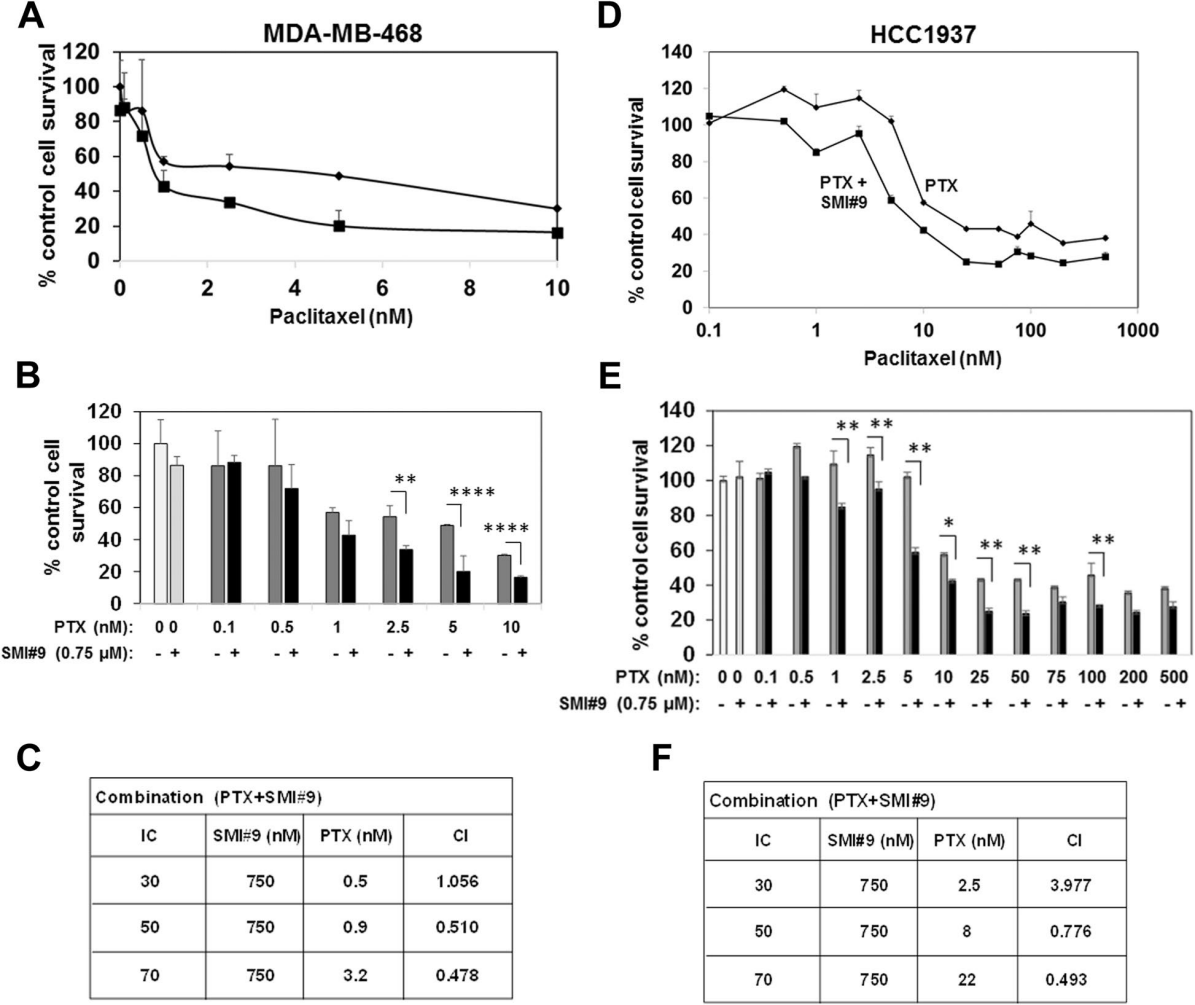
SMI#9 synergistically enhances paclitaxel (PTX) sensitivity in BRCA1 wild type and mutant TNBC cell lines**.** MDA-MB-468 (**A-C**) and HCC1937 (**D-F**) cells were treated with PTX alone at the indicated concentrations or along with 0.75 µM SMI#9 and proliferating cells measured by MTT assays. **C, F** Combination indices (CI) were calculated with the CompuSyn software where a CI > 1 indicates antagonism, CI < 1 indicates synergy, and CI = 1 indicates an additive effect. Results are expressed as mean ± S.E.M from three independent experiments. *P < 0.05, **P < 0.005, ****P < 0.00005

To assess the impact of RAD6 inhibition on PTX response, BRCA1 wild type MDA-MB-468 and BRCA1 mutant HCC1937 cells were treated with 0.1–1000 nM PTX with or without 0.75 µM SMI#9 (IC10 dose). Single treatment with 0.75 μM SMI#9 marginally decreased MDA-MB-468 cell survival compared to control whereas HCC1937 cells were unaffected. Incorporation of SMI#9 enhanced PTX sensitivities of both MDA-MB-468 (Fig. 2A-C) and HCC1937 (Fig. 2D-F) cells. Combination index (CI) analysis revealed PTX and SMI#9 synergy at PTX doses > 0.5 nM in MDA-MB-468 (Fig. 2C) and > 5 nM in HCC1937 cells (Fig. 2F).

### SMI#9 treatment decreases colony survival and enhances abnormal mitosis in BRCA1 wild type and mutant TNBC cells

**Fig. 3:**
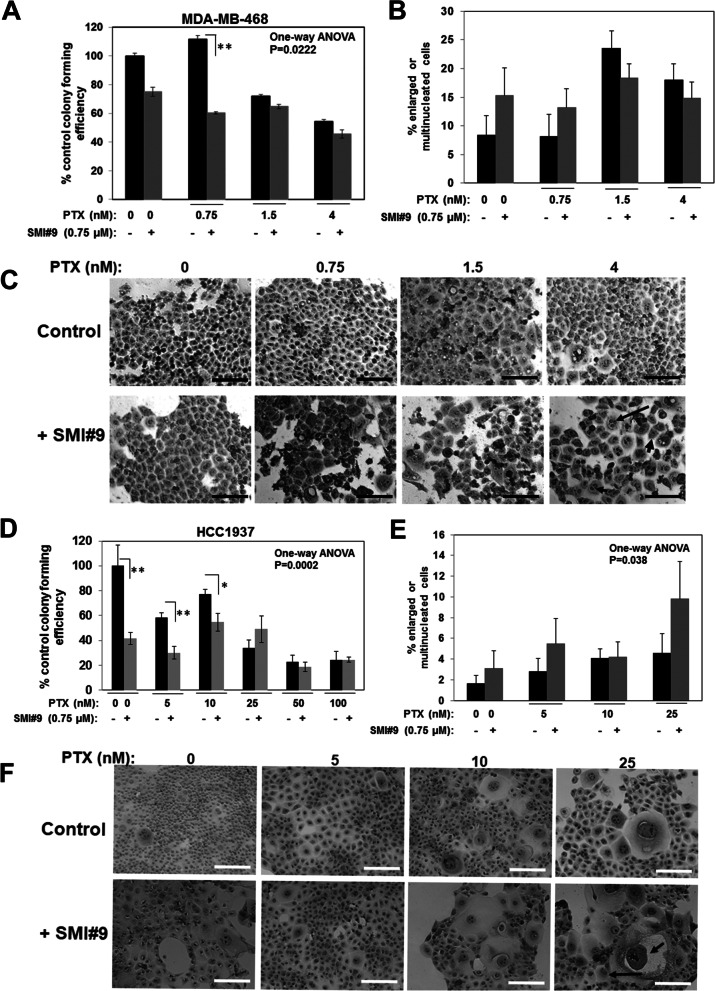
RAD6 inhibition induces multinucleation and giant nuclei formation**.** MDA-MB-468 (**A-C**) and HCC1937 (**D-F**) cells were treated with the indicated doses of paclitaxel (PTX) for 24 h, and 100 viable cells were replated as single cells in quadruplicates post PTX treatment. Colonies were allowed to form and were then either left untreated or treated with 0.75 µM SMI#9. **A**,** D** Colony forming efficiency. **A** **P < 0.005 and one-way ANOVA, *P* = 0.022; **D** *P < 0.01, **P < 0.005 and one-way ANOVA, *P* = 0.0002. **B, E** Quantitation of colonies containing giant or multinucleated abnormal cells. Results are expressed as mean ± S.E.M. of replicates. **E** One-way ANOVA, *P* = 0.038. **C, F** Representative phase contrast images (long arrow, multinucleated cells; short arrow, cell with giant nucleus). Scale bars, 20 μM

To corroborate the MTT assay data in Fig. 2, clonogenic survival assays were performed. MDA-MB-468 and HCC1937 cells pretreated with PTX (MDA-MB-468: 0.75, 1.5 and 4 nM; HCC1937: 5, 10, 25, 50 and 100 nM) were reseeded as 100 single cells and allowed to form colonies of ~ 50 cells prior to treatment with 0.75 µM SMI#9. This approach parallels the therapeutic setting as cells capable of producing colonies represent potential resistant outgrowths and allows for analyzing the impact of RAD6 inhibition on PTX resistant subpopulations. Colonies were scored 72 h post SMI9 treatment for colony forming efficiency and were microscopically analyzed for their composition (presence of enlarged or multinucleated cells, a characteristic of mitotic arrest or catastrophe). Although SMI#9 treatment significantly reduced colony forming efficiencies in both MDA-MB-468 (Fig. 3A; one-way ANOVA *P* = 0.0222) and HCC1937 (Fig. 3D; one-way ANOVA *P* = 0.0002) cells, the surviving SMI#9 treated colonies contained significantly increased numbers of cells containing giant (short arrow in Fig. 3F) or multinucleated (long arrow in Figs. 3C and F) cells that were more pronounced in HCC1937 colonies (Fig. 3B, C and E, F).

### PTX and SMI#9 treatments decrease TAU isoform expression and modulate cyclin B1 levels consistent with G2/M arrest or mitotic catastrophe

**Fig. 4:**
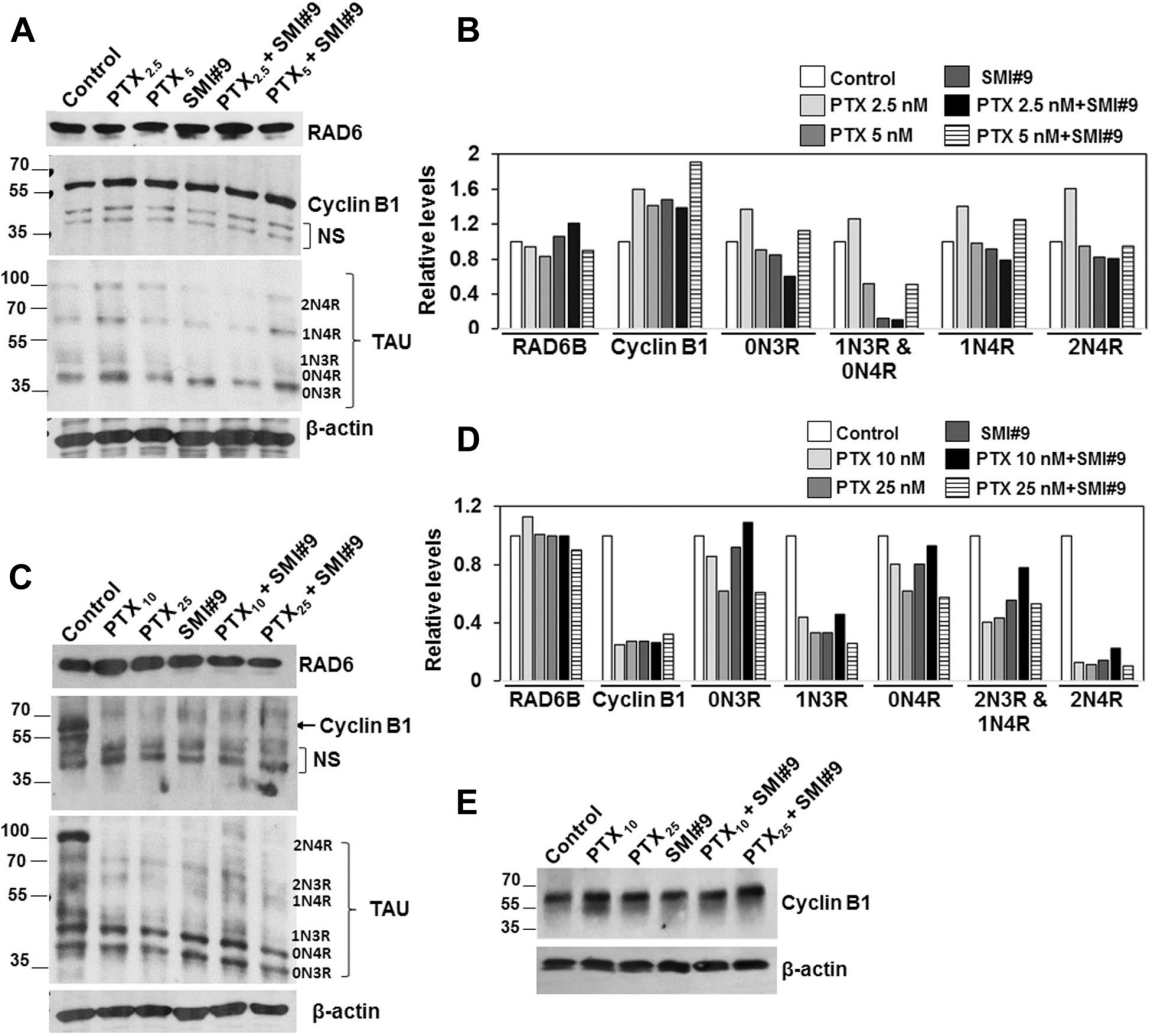
Paclitaxel and SMI#9 decrease 1N3R TAU isoform and modulate cyclin B1 protein levels differently in paclitaxel-sensitive and intrinsically resistant TNBC cells**.** Western blot analysis of the indicated proteins in MDA-MB-468 (**A, B**) and HCC1937 (**C-E**) cells treated with PTX, SM#9 or SMI#9 + PTX at the indicated nanomolar concentrations. **E** Cyclin B1 analysis in MG132 treated HCC1937 cells. **B, D** Protein levels were normalized to β-actin using Image J software

To assess the functional impact of RAD6 inhibition on PTX response, MDA-MB-468 or HCC1937 cells were treated with PTX (2.5 or 5 nM, MDA-MB-468 or 10 or 25 nM, HCC1937) with or without 0.75µM SMI#9 or vehicle, and whole cell lysates were analyzed by western blotting for expression of RAD6, TAU and cyclin B1 proteins. No discernible alterations in RAD6 protein levels resulted from PTX, SMI#9 or PTX/SMI#9 combination treatments of MDA-MB-468 or HCC1937 cells (Fig. 4A-D). However, similar treatments produced distinct effects on cyclin B1, a marker of G2/M arrest, in MDA-MB-468 and HCC1937 TNBC cells. Cyclin B1 steady state levels were increased ~ 1.5-fold in PTX or SMI#9 treated MDA-MB-468 cells as compared to control, and PTX/SMI#9 combination treatment induced 1.9-fold increase in cyclin B1 levels as compared to control (Fig. 4A, B). In HCC1937 cells, treatment with PTX, SMI#9 or PTX/SMI#9 combination caused loss of cyclin B1 as compared to control (Fig. 4C, D), a feature that is consistent with mitotic catastrophe [45] and supported by the data in Fig. 3F that showed induction of nonreproductive cells with enlarged or multiple nuclei. Treatment with nontoxic concentration of proteasome inhibitor resulted in restoration of cyclin B1 in PTX and SMI#9 treated HCC1937 cells confirming proteasome mediated destruction of cyclin B1 (Fig. 4E). Western blot analysis of TAU proteins revealed 5 of the six or all six TAU isoforms in MDA-MB-468 and HCC1937 cells, respectively (Fig. 4A and C). Consistent with SMI#9 induced PTX sensitization, among the 5 isoforms bands detected in control MDA-MB-468 cells only bands corresponding to the 1N3R and to a lesser extent the 0N4R TAU isoforms were susceptible to SMI#9 and SMI#9 + PTX treatments (Fig. 4A, B). Due to their close spacing, the 1N3R and 0N4R bands were quantitated collectively (Fig. 4B). In PTX-resistant HCC1937 cells, all six TAU isoforms were more strongly detected as compared to MDA-MB-468 cells (Fig. 4C), and treatment with PTX and SMI#9 consistently downregulated 1N3R and 2N4R TAU isoforms (Fig. 4C, D). Since the 1N3R isoform displayed susceptibility to PTX and SMI#9 in both TNBC models, our data suggest an important role for this TAU isoform in modulation of PTX and SMI#9 induced responses.

**Fig. 5:**
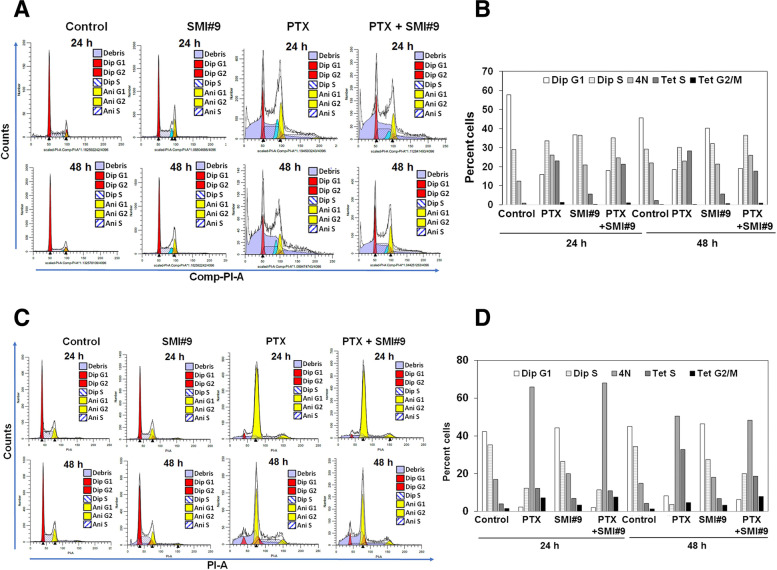
Paclitaxel and SMI#9 induce accumulation of tetraploid cells in S and G2/M phases**.** DNA content analyzed by flow cytometry after 24 and 48 h of treatment with PTX (5 nM for MDA-MB-468 and 10 nM for HCC1937 cells), SMI#9 (0.75 µM) or a combination of SMI#9 with the corresponding PTX dose. **A, B** MDA-MB-468 and (**C, D**) HCC1937 cells. **B, D** Percentage of cells in diploid and tetraploid G1, S and G2/M phases were estimated after analysis using Modfit software

To determine the cell cycle profiles in PTX, SMI#9 and PTX + SMI#9 treated MDA-MB-468 and HCC1937 cells, DNA content was evaluated using flow cytometry. Cell cycle analysis showed that PTX and SMI#9 treatments induced transition to tetraploid state in both MDA-MB-468 and HCC1937 cells. In MDA-MB-468 cells, ~ 20% of cells had 4 N DNA content in all groups including controls suggesting that generation of this population is not drug induced but an inherent property of this cell line. PTX and SMI#9 treatments of MDA-MB-468 cells increased > 25- and sevenfold, respectively, the proportion of tetraploid cells in S phase with detectable increases in G2/M phase as compared to controls (Fig. 5A, B and Supplementary Fig. 1). Unlike in MDA-MB-468 cells, in HCC1937 population cells with 4 N DNA content (tetraploid G1 state) were robustly increased by PTX and comprised > 50% of the population. PTX treatment induced a 4- and threefold increase in cells with 4 N DNA content at 24 h and 48 h, respectively, as compared to controls. Although SMI#9 treatment had only a minor effect in accumulating 4 N cells, the PTX-induced responses were maintained in PTX + SMI#9 treated cells. The proportion of tetraploid cells in S phase were also increased ~ 3 and 1.75 fold, respectively, in PTX and SMI#9 treated HCC1937 cells compared to controls, while the tetraploid cells in G2/M phase increased ~ 5 and twofold, respectively, in PTX and SMI#9 treated cells at 24 h, and ~ sixfold in PTX + SMI#9 treated cells at 48 h of treatment (Fig. 5C, D and Supplementary Fig. 1). These data reveal differences in PTX/SMI#9 induced effects on cell cycle between MDA-MB-468 and HCC1937 cells, and support the concordance between cyclin B1 loss and accumulation of 4 N (tetraploid G1 state), a feature of mitotic slippage in HCC1937 cells.

### Inhibition of RAD6 induces monopolar mitotic spindles

**Fig. 6:**
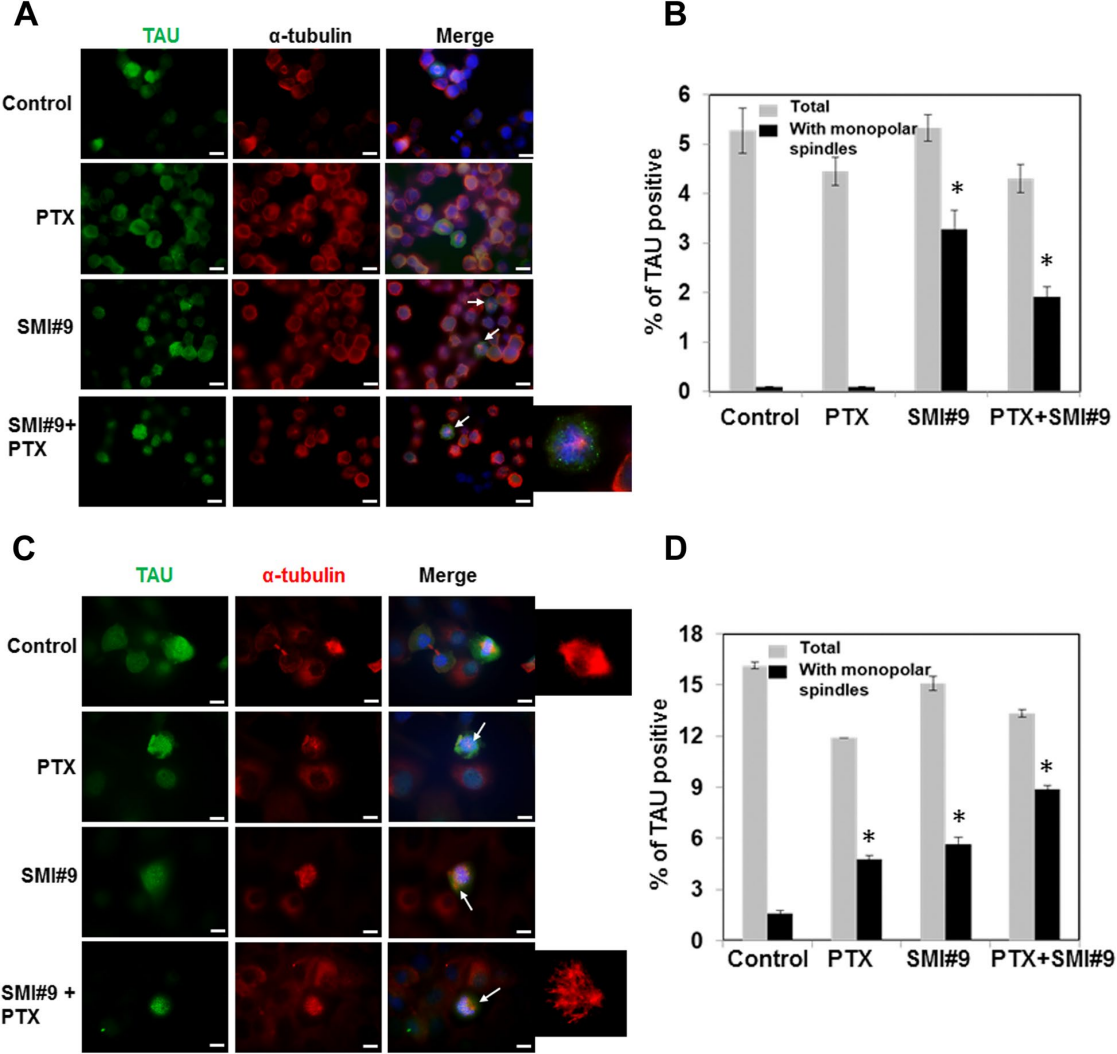
SMI#9 induces monopolar mitotic spindles**.** MDA-MB-468 (**A, B**) and HCC1937 (**C, D**) TNBC cells were treated with 2.5 or 10 nM PTX, respectively, 0.75 µM SMI#9 or SMI#9 + PTX and analyzed for TAU (green) and α-tubulin (red) expression/localization by immunofluorescence staining. Bar, 10 µm. Enlarged images of α-tubulin stained mitotic spindles are shown. **B, D** Quantitation of total TAU positive cells and those with monopolar mitotic spindles. Results are expressed as mean ± S.E.M of replicates. **B** *P < 0.05, **C** *P < 0.01

Since SMI#9 treatment increased PTX sensitivity and similarly affected 1N3R TAU isoform levels in both MDA-MB-468 and HCC1937 cells, we examined the effects of SMI#9, PTX or SMI#9/PTX combination on TAU expression and localization in mitotic cells by immunofluorescence staining using anti-TAU and anti-α-tubulin antibodies. Mitotic cells were selectively marked by intense TAU staining and treatments with PTX, SMI#9, or SMI#9 + PTX did not affect TAU localization to the mitotic spindles. Instead, SMI#9 treatment either alone or in combination with PTX significantly induced monopolar mitotic spindles in both BRCA1 wild type (Fig. 6A, B; *P* < 0.05) and BRCA1 mutant (Fig. 6C, D; *P* < 0.01) TNBC cells. Treatment with PTX alone induced monopolar mitotic spindles only in HCC1937 cells which was significantly increased further by SMI#9 combination treatment (Fig. 6C, D; *P* < 0.01). Since MDA-MB-468 cells are more sensitive to PTX than HCC1937 cells, we posit that this heightened PTX sensitivity induces cytotoxicity without accumulation of cells with aberrant mitosis. Since MDA-MB-468 cells show greater sensitivity to SMI#9 [38], we propose that SMI#9-induced sensitization may partly result from monopolar mitotic spindle induction, a feature consistent with ensuing aberrant mitotic exit that usually leads to cell death [46]. These data suggest an important role for RAD6 in MT assembly potentially via its function in centrosome regulation.

### SMI#9 treatment reverses acquired PTX resistance

**Fig. 7:**
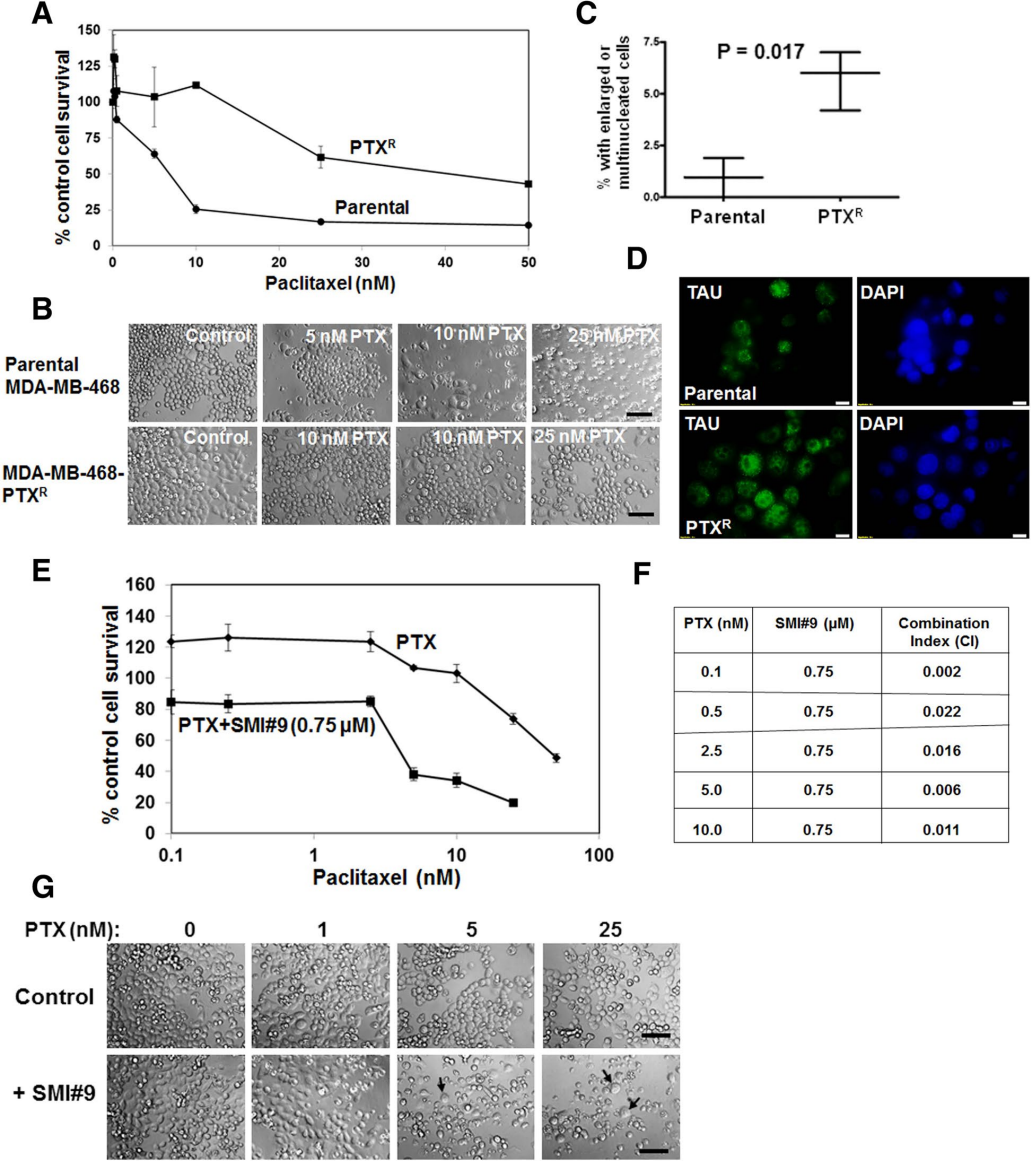
SMI#9 reverts paclitaxel (PTX) resistance. **A** PTX sensitivities of isogenic parental PTX-resistant MDA-MB-468 cells. **B** Representative phase-contrast micrographs. Original magnification × 20. **C** Quantification of cells with enlarged or multiple nuclei. Data expressed as mean ± S.E.M. **D** Immunofluorescence staining of TAU. Bar, 10 µm. **E** MDA-MB-468 PTX^R^ cells were treated with various doses of PTX with or without 0.75 µM SMI#9 and proliferating cells measured by MTT assays. Data are expressed relative to control cells as mean ± S.E.M. G. Representative phase-contrast images are shown. Cells with multiple or giant nuclei are indicated by arrow. Scale bar, 10 μM. **F** Combination indices calculated with the CompuSyn software where a CI > 1 indicates antagonism, CI < 1 indicates synergy and CI = 1 indicates an additive effect

Our data thus far showed that the RAD6 inhibitor SMI#9 increases PTX sensitivity by interfering with and by strengthening PTX-induced interference of MT dynamics. To confirm whether SMI#9 treatment can indeed reverse PTX resistance, we developed an isogenic TNBC model of acquired PTX resistance using MDA-MB-468 cells. Compared to the parental MDA-MB-468 cells (IC50 ~ 7 nM), its PTX-resistant isogenic counterpart MDA-MB-468-PTX^R^ cells demonstrate ~ 5.5-fold increase in PTX tolerance (IC50 ~ 40 nM; Fig. 7A). Phase-contrast microscopy revealed ~ threefold increase in the number of multinucleated cells (Fig. 7B, C; *P* = 0.017). Immunofluorescence analysis showed robust nuclear TAU expression excluding nucleoli in ~ 75% of MDA-MB-PTX^R^ cells as compared to ~ 30% in the parental counterpart as well as the presence of cells with numerous prominent nucleoli and enlarged nuclei in the PTX^R^ counterpart (Fig. 7D). To analyze the effect of RAD6 inhibition on PTX sensitivity, MDA-MB-468-PTX^R^ cells were exposed to increasing concentrations of PTX in the presence or absence of 0.75 µM SMI#9. MTT assays showed that incorporation of SMI#9 with PTX dramatically decreased PTX IC50 of MDA-MB-468-PTXR cells to 4.4 nM, restoring PTX sensitivity to that of its parental counterpart (Fig. 7E). Combination index analysis confirmed the synergism between SMI#9 and PTX (Fig. 7F). As observed with HCC1937 cells (Fig. 3E, F), cotreatment of MDA-MB-PTX^R^ cells with PTX and SMI#9 increased the proportion of cells with enlarged and multiple nuclei (Fig. 7G, arrows). These data reveal concordance in SMI#9 regulation of PTX sensitivities in TNBC cells with intrinsic or acquired PTX resistance and implicate RAD6 as a potential therapeutic target for alleviating PTX resistance.

## Discussion

Our findings support an important role for RAD6B in mitotic spindle dynamics and centrosome function as it is the major RAD6 homolog overexpressed in breast cancer cells and suggest that inhibition of RAD6B could be applied to increase PTX sensitivities of BRCA1 wild type and mutant TNBC cells. We also show that while all four TNBC models used in this study show centrosome amplification regardless of their BRCA1 status, only the PTX sensitivities of the basal subtype TNBC cell models correlated with their BRCA1 status as BRCA1 mutant HCC1937 cells were more resistant to PTX as compared to BRCA1 wild type MDA-MB-468 cells whereas the PTX sensitivities of the mesenchymal subtype (MDA-MB-231 and SUM1315) showed no such correlation. Consistent with overexpression of RAD6B in both BRCA1 wild type and mutant TNBCs [36, 38], SMI#9 treatment enhanced PTX sensitivities of both MDA-MB-468 and HCC1937 cells and this increase in PTX sensitivity was accompanied by increases in nonreproducing cells with enlarged or multiple nuclei (a characteristic of mitotic catastrophe induction) which was more pronounced in HCC1937 cells. Using the isogenic MDA-MB-468 model of acquired PTX resistance (MDA-MB-468-PTX^R^), we demonstrated that SMI#9 treatment can restore PTX sensitivity, and as with PTX-resistant HCC1937 cells, SMI#9 synergized with PTX and similarly increased cells containing giant nuclei.

Mitotic catastrophe, a tumor-suppressive mechanism, is triggered during or after aberrant mitosis as a result of DNA damage or deranged spindle formation and defective G2 checkpoint that arrest mitotic progression. Mitotic catastrophe can produce giant or multinucleated aneuploid cells that undergo cell death or remain metabolically active but lack reproductive potential. Since SMI#9 and PTX treatments caused increases in cells with giant nuclei, we assessed their effects on microtubule associated protein TAU and cyclin B1. Whereas PTX-sensitive MDA-MB-468 cells showed increases in cyclin B1 levels with PTX that were enhanced by SMI#9, HCC1937 cells treated with PTX, SMI#9 or combination expressed undetectable levels of cyclin B1, indicating that the cells are not undergoing a typical G2 arrest. Premature degradation of cyclin B1 is linked to mitotic slippage, which results from the release of G2/M arrested cells that have not satisfied the spindle assembly checkpoint [45–47]. Mitotic cyclin B1 destruction induce the 4 N cells to enter a tetraploid G1 state after mitotic slippage caused by prolonged drug-induced mitotic blocks. This results in cells with increased DNA content/multinucleation that either exit mitosis and continue to cycle, undergo cell death within G1, or remain viable but reproductively dead [46–48]. Consistent with PTX/SMI#9 induced cyclin B1 loss in HCC1937 cells, cell cycle analysis revealed accumulation of cells with 4 N DNA content, a feature of mitotic slippage. Interestingly, unlike in HCC1937 cells, drug treatment did not dramatically increase MDA-MB-468 cells with 4 N DNA content compared to controls, confirming that induction of 4 N-G1 state ensues from cyclin B1 loss. It remains to be determined whether PTX induced cyclin B1 loss is dependent on *BRCA1* status. Previous reports have shown that taxol [49] and docetaxel [50] similarly increase cyclin B1 levels in BRCA1 wild type MDA-MB-231 cells and MDA-MB-468 cells suggesting that cyclin B1 loss may not be unique to PTX resistant TNBC cells. SMI#9 treatment increased accumulation of tetraploid cells in G2/M phases, suggesting that addition of SMI#9 enhances PTX chemosensitivity by strengthening G2/M arrest and cell death after mitotic slippage. The results from clonogenic survival assays also suggest that PTX may induce a leaky G2/M arrest in HCC1937 cells that allows for cell cycle continuation, and that combination with SMI#9 may strengthen the durability of this arrest as evidenced by increases in cells with giant or multiple nuclei and subsequent loss of cell viability. Since SMI#9 treatment of MDA-MB-468-PTX^R^ cells similarly increases cells containing enlarged or multiple nuclei, our data suggest that inhibition of RAD6B may offer a new strategy to strengthen cell cycle arrest and decrease reproductive potential.

TAU is a MT-associated protein that functions to reversibly stabilize microtubules. TAU preferentially binds to assembled MTs in the same region as PTX [13, 51], and this binding can set off a competition between TAU and PTX for binding to MTs [51–54]. TAU overexpression has been associated with reduced PTX efficacy in metastatic breast cancer patients [52, 53, 55–57]. Our data showed that the 1N3R TAU isoform is downregulated by SMI#9 and PTX treatments in both MDA-MB-468 and HCC1937 cells, whereas the 2N4R TAU isoform was only lost in PTX and SMI#9 treated HCC1937 cells. The underlying reasons for 2N4R TAU susceptibility to PTX or SMI#9 remains to be determined as it is the largest TAU containing additional amino terminal inserts and an extra MT binding repeat and possessing stronger microtubule binding and microtubule stabilizing activities as compared to 3R TAU isoforms [58, 59]. Since the 1N3R isoform is decreased by PTX and SMI#9 in both TNBC models, our data suggest a common mechanism of 1N3R regulation by SMI#9 and PTX. It is possible that the other TAU isoforms may bind at different sites or may bind more tightly to MTs and require higher drug concentrations for their displacement [59]. Abnormal release of TAU from MTs has been shown to increase their affinity for protein misfolding and degradation by the proteasome [60–62], supporting the possibility that SMI#9 or PTX displacement of 1N3R TAU promotes its degradation. Immunofluorescence analysis indicated that nuclear TAU expression was not affected by PTX or SMI#9 treatments. Since the TAU antibody used in our study recognizes all six isoforms, we speculate that loss of SMI#9 and PTX-sensitive 1N3R (and 2N4R) isoforms may be masked by the continued presence of the other isoforms. Formation of a bipolar spindle apparatus involves motor proteins and controlled MT polymerization and depolymerization, which in turn is required for faithful chromosome segregation. However, defects in centrosome proteins, motor proteins or kinases induce the assembly of aberrant MT spindles with monopolar morphology or with poorly separated poles. Induction of monopolar spindles is considered a useful strategy for cancer therapy since the ensuing aberrant mitotic exit typically results in cell death [46]. In this context, the induction of monopolar mitotic spindles by SMI#9 and PTX not only support a common potentially TAU-mediated mechanism of MT dynamics regulation but also indicate their potential value in cancer therapy.

**Fig. 8:**
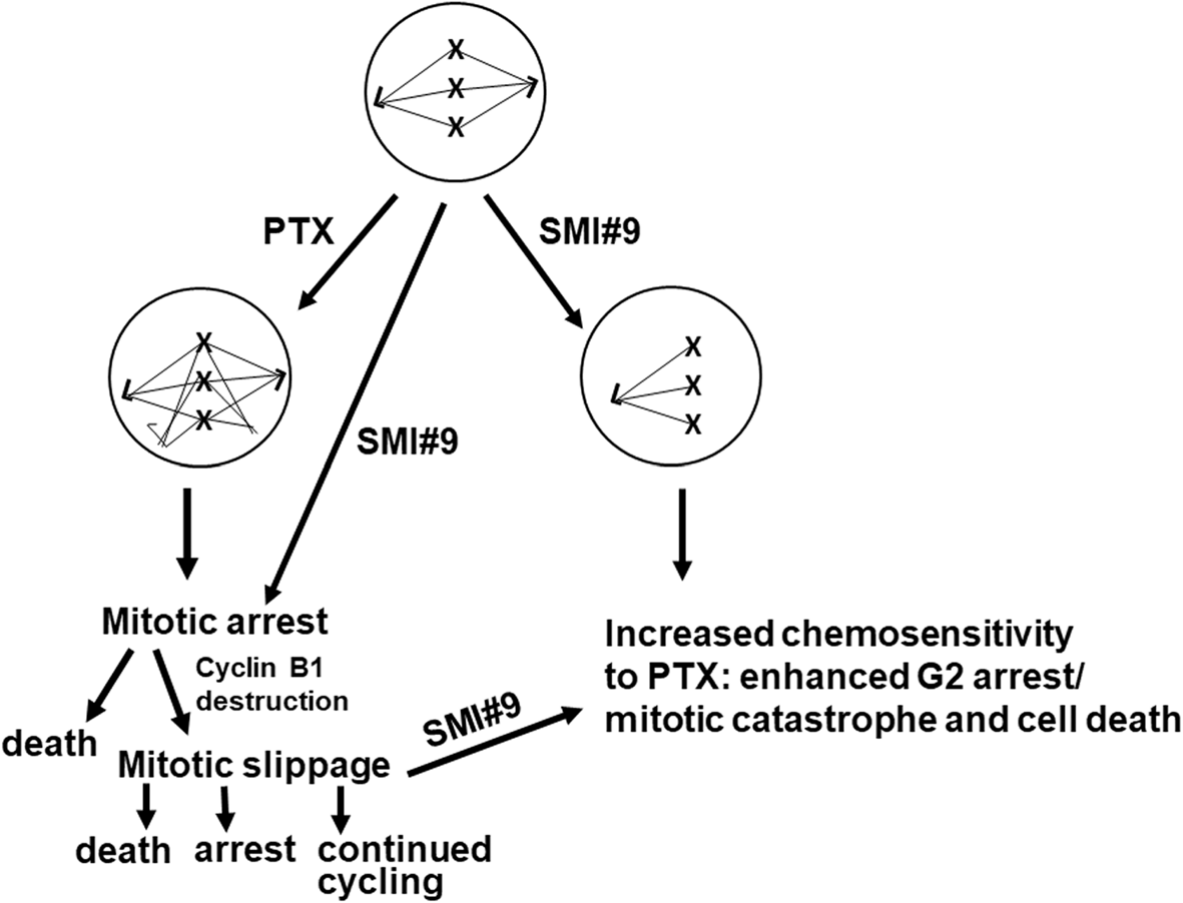
A schematic diagram of working mechanism of SMI#9/PTX combination therapy in TNBC chemosensitization**.** PTX or SMI#9 treatments induce mitotic arrest which can result in cell death or mitotic slippage. Formation of tetraploid G1 cells, a feature of mitotic slippage, is facilitated by premature cyclin B1 destruction and abnormal mitotic exit without chromosome segregation or cytokinesis. SMI#9 treatment enhances PTX cytotoxicity by inducing monopolar mitotic spindles and strengthening arrest and mitotic catastrophe of cells that slipped out of prolonged mitotic arrest

Using an isogenic TNBC model of acquired PTX resistance as well as TNBC models with inherent differences in PTX sensitivities, we show that treatment with RAD6 inhibitor SMI#9 enhances PTX sensitivities. While PTX-resistant HCC1937 or MDA-MB-PTX^R^ cells contain subpopulations with giant or multiple nuclei that may provide a mechanism of PTX resistance, our data suggest that SMI#9 can enhance PTX sensitivity and cell death by inducing monopolar spindles and strengthening the durability of cell cycle arrest and mitotic catastrophe. Whereas SMI#9 therapeutic activity on TNBC models has been demonstrated in vivo [36, 38, 40], translation of the in vitro SMI#9/PTX interaction data awaits in vivo confirmation which is underway. The results obtained above are summarized schematically in Fig. 8.

## Conclusions

Taken together, our data suggest that induction of monopolar spindles, and loss of cyclin B1 and 1N3R TAU isoform may be used as markers for assessing TNBC PTX sensitivity. Our data also provide mechanistic support for the role of RAD6B in centrosome function that is critical for completion of mitosis and suggest that inhibition of RAD6B may be therapeutically beneficial for patients with BRCA1 wild type or mutant TNBCs treated with PTX.

## Supplementary Information

**Additional file1:** **Supplementary Fig. 1.** Graphical representation of the percentage of MDA-MB-468 and HCC1937 cells in tetraploid G1, S and G2/M phases following treatment with PTX, SMI#9 or PTX+SMI#9 compared to controls. **Supplementary Fig. 2.** Figure 4A (MDA-MB-468) and Figure 4C (HCC1937) western blots of cyclin B1, Tau, Rad6 and β-actin. Figure 4E (western blot of cyclin B1 and β-actin in HCC1937 treated with MG132).

## Data Availability

All data generated during this study are included in this article and its supplementary information files.

## Abbreviations

PTX: Paclitaxel
TNBC: Triple negative breast cancer
γ-TuRCs: γ-Tubulin ring complexes
MT: Microtubules
TLS: Translesion synthesis
TCGA: The Cancer Genome Atlas
CI: Combination index
MTT: 3-(4,5-Dimethylthiazol-2-yl)-2,5-diphenyl-2H-tetrazolium bromide
