# Supplementary material for: RAD6 inhibition enhances paclitaxel sensitivity of triple negative breast cancer cells by aggravating mitotic spindle damage
Source: BMC Cancer. 2022 Oct 18;22:1073. doi: 10.1186/s12885-022-10119-z (PMC9578210; doi:10.1186/s12885-022-10119-z)

**Supplementary Fig. 1.** Graphical representation of the percentage of MDA-MB-468 and HCC1937 cells in tetraploid G1, S and G2/M phases following treatment with PTX, SMI#9 or PTX+SMI#9 compared to controls.

**Supplementary Fig. 2.**

**Figure 4A (MDA-MB-468) and Figure 4C (HCC1937) western blots of cyclin B1, Tau, Rad6 and β-actin.**

**
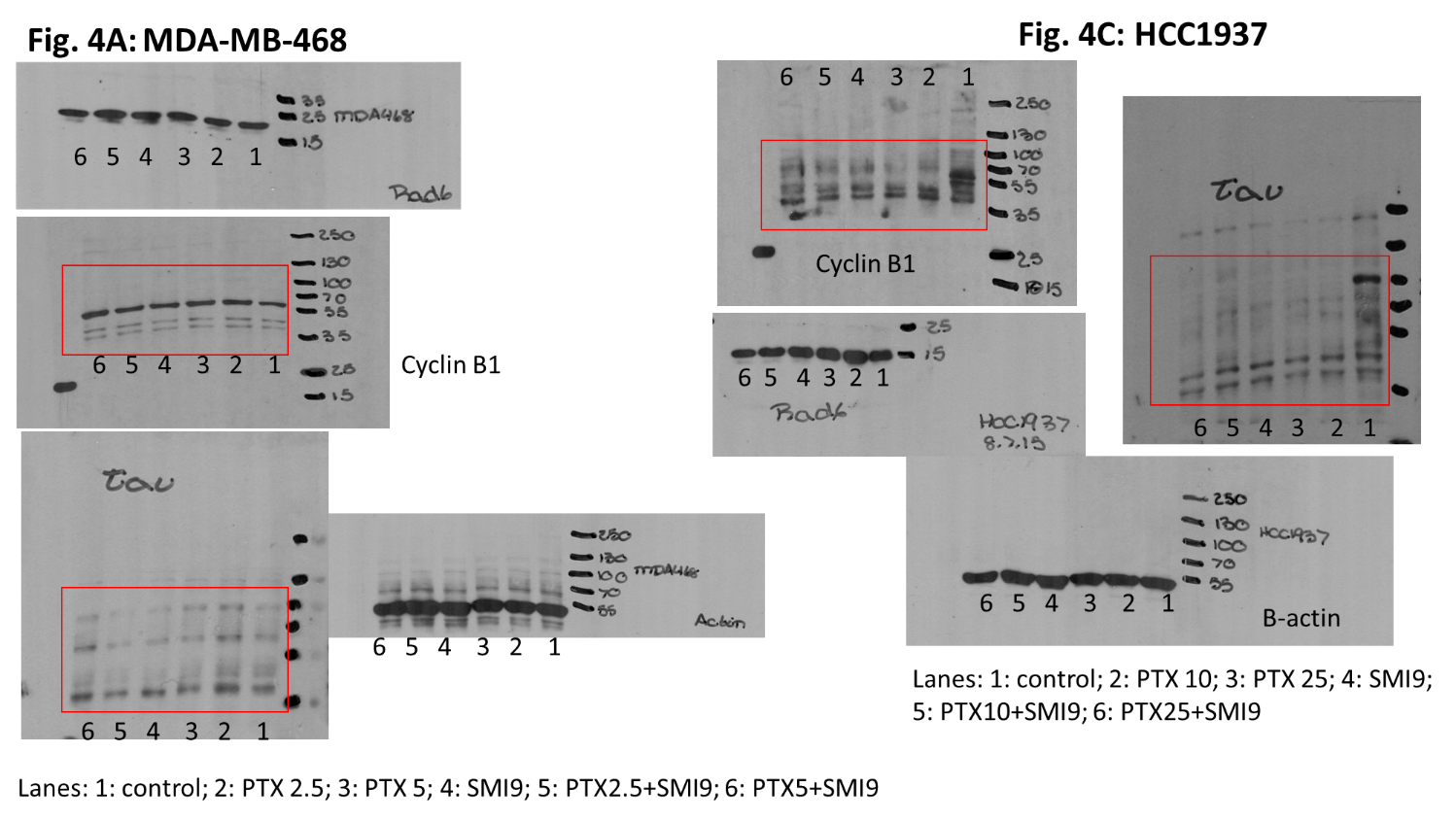
**

**Figure 4E (western blot of cyclin B1 and β-actin in HCC1937 treated with MG132)**


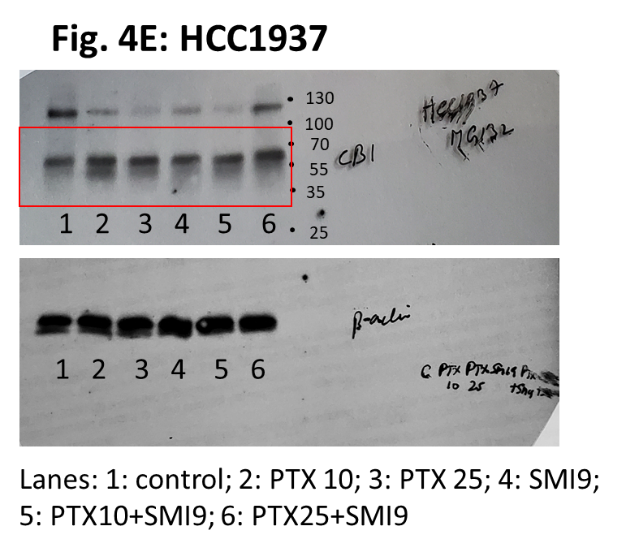

Supplement: Supplementary file 1 — Additional file1: Supplementary Fig. 1. Graphical representation of the percentage of MDA-MB-468 and HCC1937 cells in tetraploid G1, S and G2/M phases following treatment with PTX, SMI#9 or PTX+SMI#9 compared to controls. Supplementary Fig. 2. Figure 4A (MDA-MB-468) and Figure 4C (HCC1937) western blots of cyclin B1, Tau, Rad6 and β-actin. Figure 4E (western blot of cyclin B1 and β-actin in HCC1937 treated with MG132). [file 12885_2022_10119_MOESM1_ESM.docx]
